# A New Model of Esophageal Cancers by Using a Detergent-Free Decellularized Matrix in a Perfusion Bioreactor

**DOI:** 10.3390/bioengineering10010096

**Published:** 2023-01-11

**Authors:** Jordan Brennan, Michael L. Lu, Yunqing Kang

**Affiliations:** 1 Department of Ocean and Mechanical Engineering, College of Engineering and Computer Science, Florida Atlantic University, Boca Raton, FL 33431, USA; 2 Department of Biomedical Science, Charles E. Schmidt College of Medicine, Florida Atlantic University, Boca Raton, FL 33431, USA; 3 Faculty of Integrative Biology PhD Program, Department of Biological Science, Florida Atlantic University, Boca Raton, FL 33431, USA

**Keywords:** decellularization, esophageal cancer, dynamic culture, cancer model

## Abstract

The lack of physiologically relevant human esophageal cancer models has as a result that many esophageal cancer studies are encountering major bottleneck challenges in achieving breakthrough progress. To address the issue, here we engineered a 3D esophageal tumor tissue model using a biomimetic decellularized esophageal matrix in a customized bioreactor. To obtain a biomimetic esophageal matrix, we developed a detergent-free, rapid decellularization method to decellularize porcine esophagus. We characterized the decellularized esophageal matrix (DEM) and utilized the DEM for the growth of esophageal cancer cell KYSE30 in well plates and the bioreactor. We then analyzed the expression of cancer-related markers of KYSE30 cells and compared them with formalin-fixed, paraffin-embedded (FFPE) esophageal squamous cell carcinoma (ESCC) tissue biospecimens. Our results show that the detergent-free decellularization method preserved the esophageal matrix components and effectively removed cell nucleus. KYSE30 cancer cells proliferated well on and inside the DEM. KYSE30 cells cultured on the DEM in the dynamic bioreactor show different cancer marker expressions than those in the static well plate, and also share some similarities to the FFPE-ESCC biospecimens. These findings built a foundation with potential for further study of esophageal cancer behavior in a biomimetic microenvironment using this new esophageal cancer model.

## 1. Introduction

Esophageal cancer is currently the sixth most common cancer worldwide and one of the most lethal [1], due to the lack of early detection methods [2] and effective treatment [3].

Most studies on esophageal cancer phenotype, aggressiveness, metastasis, or drug discovery are commonly performed in two-dimensional (2D) well plates or in xenograft animal models [4,5,6]. A 2D cell culture lacks sufficient 3D esophageal biological tubular structure and microenvironment [7,8,9]. Tumor xenograft animal models do mimic in vivo tumor microenvironments. However, most of the xenograft animal ectopic models do not adequately represent the specific requirements for the in vivo development of esophageal cancers [10,11], and there is a gradual advancement towards reducing or eliminating animal testing in search of alternatives. Furthermore, animal models yield data that may not reflect the biological outcome or pathways associated with human tissues [12]. These difficulties have impeded the establishment of an animal orthotopic esophageal tumor model. 

Therefore, there is increasing interest in the development of a 3D in vitro culture model for bridging the research gap between 2D culture and animal xenogeneic models [13,14]. Presently, there are efforts to develop 3D esophageal cancer models to replicate the tumor-specific cellular and matrix microenvironments based on the tissue engineering concept. However, although there are many 3D models under development to study cancer, a consistent theme is varying efficacy and approach [15,16,17], and they are rarely derived from a living tissue-specific source. For example, some use synthetical porous scaffolds for investigating tumor tissue [18]. There are also hydrogel cancer spheroid models [15], microfluidics applications for modelling cancer [17], 3D-printed models [19], and spheroid in well plate models [20]. Those models are not as relevant to human esophageal extracellular matrix (ECM) as a faithfully native-derived esophageal ECM prepared for cell seeding. Scaffolds used in these models may not represent a biologically active ECM, which limits the ability of the cancer cells to interact with native matrix components, potentially limiting the manifestation of cancer cell behavior. As their structure and composition are far different from esophageal ECM, these synthetic ECMs for esophageal cancer cells cannot fully represent the native in vivo esophageal tumor matrix microenvironment.

An appropriate tissue-derived decellularized matrix will offer a close resemblance to tissue matrix in vivo to support the cancer cells to interact in a more native environment. A native tissue-derived decellularized matrix may promote the expression of specific cell phenotypes, which synthetic scaffolds are incapable of reproducing [21,22]. However, most currently established decellularization methods utilize harsh detergents and have lengthy decellularization processes with inconsistent results. Many methods utilize toxic or harsh chemicals, which could alter ECM properties or are difficult to manage for repeatable results [23,24,25,26,27,28,29,30,31]. It has been shown that the conventional decellularization processes employed by these methodologies may drastically denature the ECM properties that essentially affect morphogenesis, invasion, cell polarity, and other facets of cancer cell behavior [32,33,34,35], which raises a question of relevance and representation in in vivo conditions [36].

Therefore, in this study we developed a detergent-free protocol for decellularizing porcine esophageal matrix (DEM). The protocol rapidly removed cells while leaving the majority of matrix components intact in a rapid two-step procedure without any particularly specific equipment and harsh chemicals used. The goal of this study was to investigate whether the decellularized pig esophageal matrix can support the growth of esophageal squamous cell carcinoma (ESCC) under a static well plate culturing condition and a customized dynamic culture bioreactor. We evaluated the degree of biological mimicry of DEM in relation to in vivo tumors by comparing the cancer cell markers expressed on the ESCC cultured on the DEM with those markers expressed by esophageal squamous cell carcinoma tissue in formalin-fixed, paraffin-embedded (FFPE) biospecimens.

## 2. Materials and Methods

### 2.1. Materials

Hematoxylin (Epredia, 3537-16) and Eosin (Richard Allen scientific, 71204), and Masson’s trichrome (Epredia, 87019) were obtained from Fisher Scientific (Hampton, NH, USA). Primary antibodies: Fibronectin (ab23751), Laminin (ab11575) were obtained from Abcam, and Collagen-I and III (2150–2555) were from Bio-Rad (Hercules, CA, USA). DAPI (4′,6-diamidino-2-phenylindole), LIVE/DEAD Viability/Cytotoxicity Kit, Quant-iT PicoGreen dsDNA assay kit were obtained from Thermo Fisher Scientific (Waltham, MA, USA). Vectastain ABC Kit and DAB Peroxidase substrate kit (SK-4100) were from Vectorlabs (Burlingame, CA, USA). Alcian Blue was obtained from MilliporeSigma (Burlington, MA, USA) (TMS010C). DNase I enzyme was purchased from Thermo Scientific (Waltham, MA, USA) (EN0521).

### 2.2. Decellularization of Porcine Esophagus

Porcine esophagi were freshly obtained from a local slaughterhouse (Mary’s Ranch, Miami) and immediately soaked and preserved in antimycotic (Amphotericin B)/anti-biotics (Pen/Strep) PBS on ice. The outer longitudinal muscularis externa was removed mechanically to expose esophageal submucosa layers for treatment. Tissue was rinsed with distilled water as needed. Esophagus was then cut into segments of 12–18 mm length. A dilute solution of ammonium hydroxide with concentration of 2 M was added to esophagus tissue for 2–4 h with gentle agitation at 4 °C. After ammonium hydroxide (NH_3_·H_2_O) treatment, the tissue was rinsed briefly, once in distilled water. The rinsed tissue was further incubated at 4 °C for 6–8 h or until complete separation of epithelium from the matrix was achieved. Following ammonium hydroxide treatment, the epithelial cell layer should easily separate from the esophagus lumen as a single component, using mild mechanical assistance if needed. Once the epithelial layer was removed, the esophagus tissue was rinsed with distilled water several times to rinse out remaining base ammonium solution. The tissue was then buffered and incubated at 37 °C with 70 U/mL DNase I enzyme on a rotator platform for up to 48 h to remove remaining cell DNA fragments. 

### 2.3. Characterization of Decellularized Esophageal Matrix

#### 2.3.1. Decellularization

To quantify the residue of the porcine cell nuclei, a Quant-iT Pico-Green dsDNA assay kit was used. The DEM and native esophagus samples were treated in 0.2% Triton X-100 and subjected to three consecutive freeze/thaw cycles. The suspension was then sonicated and centrifuged. DNA standard solutions were prepared. Then, 50 µL of standard curve dilutions and DNA lysate samples were loaded into a 96-well plate. A quantity of 50 µL of Pico-Green was then added to each well. The plate was read at 480/520 nm (excitation/emission) on a fluorescence spectrophotometer (SpectraMax Gemini EM, Molecular Devices, San Jose, CA, USA). The amount of dsDNA was calculated by comparing the standard curves of the known dsDNA sample according to the manufacturer’s instructions.

To determine whether the porcine cell nuclei were removed, the DEM and native esophagus were stained by DAPI solution (5 µg/mL) to visually confirm the removal of cell nuclei. The stained samples were then examined using a Nikon fluorescent microscope (TE-2000). 

To further confirm the removal of the cell nucleus, hematoxylin and eosin (H&E) staining was used. DEM and native esophageal tissue were subject to a serial of dehydration in ethanol, and a paraffin embedding process was performed based on our previously established protocol. Paraffin block was mounted on a microtome, and 6 μm slides were cut and mounted onto positively charged microscope glass slides. Slides were subsequently stained by H&E and imaged. 

#### 2.3.2. Matrix Proteins

To examine whether the new decellularization method maintained the matrix components, immunohistochemical (IHC) staining on matrix proteins was performed. Slides were deparaffinized and unmasked at 98–99.8 °C using antigen retrieval buffer (Citrate buffer 10×, Scytek Laboratories HERI) for 20 min. After unmasking, 3% H_2_O_2_ was added for 10 min before being blocked with 5% bovine serum albumin for at least 1 h at room temperature (RT). After epitope retrieval and blocking, the following primary antibodies were added for incubation at least 1 h at RT: collagen I/III (1:40, 2150–2555 Bio-Rad), fibronectin (1:500, ab23751), laminin (1:300, ab11575). Afterwards, secondary anti-biotinylated binding IgG was added onto the samples and incubated at RT for 30 min. After rinsing, the samples were incubated with a working ABC solution (Vector Lab) for 5 min at RT and then with working DAB solution (Vector lab) for a few minutes until the sample stained. The sections were then counterstained with hematoxylin and then preserved by mounting medium (Fisher Scientific) with coverslips. Images were then taken using a Nikon TE-2000 microscope.

#### 2.3.3. Masson Trichrome and Alcian Staining

Slides were deparaffinized and hydrated; then, Masson trichrome staining and Alcian staining were performed according to the manufacturer’s protocols. The stained samples were then imaged by a microscope.

### 2.4. Cell Viability on the Decellularized Matrix

To examine whether the DEM supports cell growth of esophageal cancer cells, an esophageal cancer cell line, KYSE30 from Sigma-Aldrich (St. Louis, MO, USA), was used. KYSE30 was cultured in RPMI 1640 Medium (1× Gibco, ref A10491-01) with antibiotics/mycotic (Gibco, streptomycin sulfate and Penicillin G sodium/Amphotericin B, ref 15240-062) at 37 °C in a CO_2_ incubator. When cells were grown to near confluence, they were trypsinized and cell suspensions were seeded on the DEM under two culture conditions: static culture and dynamic culture. For static conditions, DEM was placed in 24-well plate and seeded with 1 × 10^5^ KYSE30 cancer cells, and media was changed every 2 days. For dynamic conditions, a 40 µL cell suspension of around 1 × 10^5^ KYSE30 cells was seeded onto the inner lumen surface of the DEM circumference. After cell seeding, DEM was rested for 2 h to allow time for cells to attach and subsequently mounted to circulation jars where culture medium was continually perfused at 15 µL/minute for the remaining duration of experiment. For the dynamic culture system, typical 1000 µL pipette tips were cut to desired lumen diameter and fit for flow orifice, then perforated using a razor blade to allow cell seeding from the sides into lumen (cut ‘windows’ into the pipette tip for cell seeding and diffusion characteristic), and flow/diffusion of culture media to extraneous feature of flow chamber. Solution passively diffused through the perforations and applied flow rate through orifice condition allows consistent nutrition and waste removal, thus enabling dynamic culture conditions with variable flow rate and as needed diameter setting, which accounted for variability in esophageal matrix.

To verify the cell viability of KYSE30 cultured on the DEM, after 7 days, a Live/Dead^®^ Viability/Cytotoxicity Kit for mammalian cells from Invitrogen was conducted. Cells/DEMs were stained with 6 μM EthD-1 and 2 μM Calcein AM and incubated at room temperature for 40 min in the dark, and then washed and stored in PBS before analysis by microscopy. Red indicates dead cells and green indicates alive cells. 

### 2.5. H&E and Alcian Blue Staining

At the end point of culture, cells/DEM were harvested from the static and dynamic culture system and fixed in paraformaldehyde. The fixed samples experienced graded dehydration and paraffin embedding, and were cut at 6 μm with a microtome into slide sections. The sections were stained by H&E staining and Alcian staining. 

### 2.6. Immunohistochemistry Staining

To investigate how KYSE30 cancer cells expressed cancer cell markers under the two conditions, immunohistochemical staining on the following cancer markers was performed on the sections following the protocol described in 2.3.2. These markers include: E-cadherin (1:400, ab40772, N-cadherin (1:400, ab98952), P63 (1:4000, ab735), periostin (1:2500, ab14041), C-met N-terminal (1:2000, ab51067), CD44 (1:250, ab189524), MMP9 (1:380, ab58803), TAZ (1:100, ab242313), and NCR1 (1:200, ab233558). Appropriate secondary antibodies from Vectorlabs were incubated with the sections. DAB peroxidase substrate kit was used following the manufacturer’s protocol. 

To compare the expression of cancer markers on the KYSE30 cultured in the static culture condition and the dynamic culture system, in this study formalin fixed paraffin embedded (FFPE) ESCC tissue blocks were obtained from AMSBIO company (Cambridge, MA, USA) and subjected to the same IHC protocols as above for comparison during the same time. Using a semi-quantitative method that compares the staining intensity to number of visible cells, we evaluated the gross appearance of immunohistochemistry staining on a staining intensity/cell-area basis, where total number of cells which express markers at some average intensity is weighted against cells which do not express any markers (if any are present) over the entire area occupied by all cells. At least eight pictures were taken on several regions of interest (ROI) from multiple samples of each group and manual cell counting was employed as needed for accuracy and validity to compare the stained intensity vs. cell-area for the nine cancer markers. With NIH ImageJ Software, a customized computation code for semi-quantitative image processing was written to generate semi-quantitative data from the images taken of ROI (code available in Supplementary Materials). The average intensity of staining on all these images versus cell-area was graphed by using GraphPad Prism (GraphPad, San Diego, CA, USA). 

### 2.7. Statistical Analysis

All experiments were carried out using triplicate (n = 3) for statistical analysis of results. A Student *t*-test was used to evaluate the statistical differences for all assays. When *p* value is less than 0.05, differences between groups will be considered significant.

## 3. Results

### 3.1. Decellularization of Esophageal Tissue

**Figure 1:**
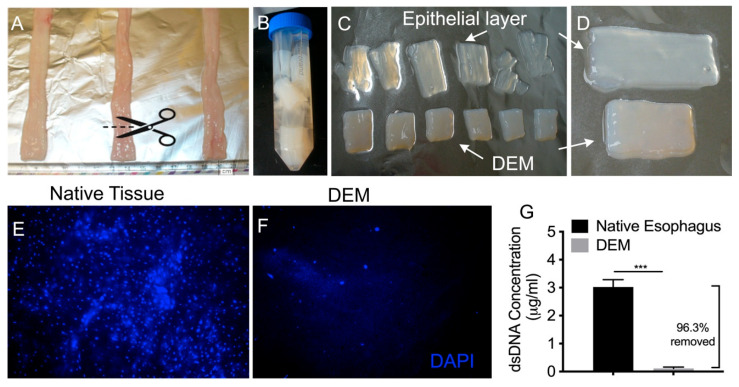
Decellularizing porcine esophagus. (**A**) Native Esophagus, wet with antibiotic/antimycotic after removing external muscle layer using scissors and blade. Cut into tubular sections suitable for flow through. (**B**) Ammonium hydroxide base decellularization; a solution of 2M ammonium hydroxide in water is added for 2 to 4 h at 4 °C (depending on thickness), followed by one brief rinse in distilled water, then incubation 4 °C for 6–8 h. (**C**) Epithelium will slide out easily. (**D**) Magnified digital pictures of the detached epithelial layer and final decellularized matrix. DAPI staining of cell nucleus of native tissue (**E**) and decellularized esophageal matrix (DEM) (**F**). (**G**) PicoGreen test showed the dsDNA concentration in the native tissue and DEM: 96.3% DNA was removed (n = 3). Asterisk “***” indicates statistically significant difference (*p* < 0.005).

In this study, we obtained fresh porcine esophagi from a local farmer (Figure 1A). The outer longitudinal muscularis externa was dissected away for decellularization. We then cut the esophagus into segments around 12–18 mm (Figure 1A). The tissue segments remained in the circular shape of a tubular esophagus. During the decellularization process, after 2M ammonium hydroxide in water without buffer was added, the esophagus started to attain a milk-like transparence (Figure 1B). The samples were rinsed once in distilled water and incubated at 4 °C overnight (6–8 h), after which the epithelial layer completely separated from the basal lamina underneath, as it gained a translucent appearance and tends to slide out of the lumen with no mechanical dissection or intervention (Figure 1C). Epithelial tissue is composed of basal, spinosum, granulosum, lucidum, and corneum layers, all of which are removed as a single epithelium layer that contains the entirety of epithelial cells, and it is necessary to remove them all in an effort to decellularize the matrix (Figure 1D). This result shows that our method allowed them to be removed intact in a single membrane sheet (Figure 1D), leaving only a sparse few residual cells which were disrupted by the alkaline ammonium water solution and subsequently decellularized in the following steps. DAPI staining reveals appearance of cell nuclei before the decellularization (Figure 1E). After decellularization, we observed an absence of cells (Figure 1F). A quantitative approach was taken; 50 µL of standard curve dilutions and DNA lysate samples were loaded into a 96-well plate. An amount of 50 µL of Pico-Green was then added to each well. The plate was read at 480/520 nm (excitation/emission) on a fluorescence spectrophotometer, which is able to detect the concentrations of double-stranded DNA fragments. The result showed that 96.3% dsDNA had been effectively removed from the DEM matrix (Figure 1G). 

### 3.2. Characterization of Decellularized Esophageal Matrix

**Figure 2:**
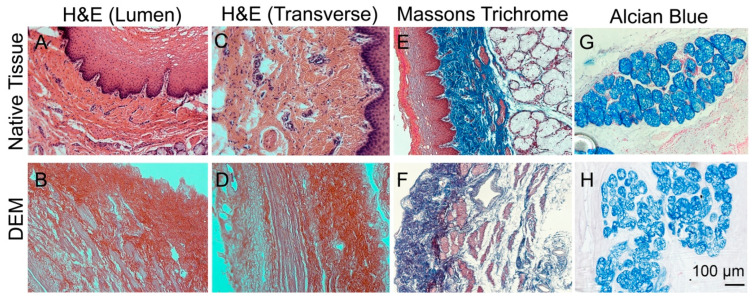
H&E, Masson’s Trichrome, and Alcian blue staining. H&E staining of native, untreated porcine esophagus (**A**,**C**) and DEM (**B**,**D**). Masson’s Trichrome staining of native tissue (**E**) and DEM (**F**). Alcian Blue staining for glycosaminoglycan (GAG) of native tissue (**G**) and DEM (**H**).

The H&E staining result further shows that native, untreated tissue exhibits intact cell layers, which are easily identifiable (Figure 2A,C) in the non-treated esophagus. After decellularization, cell nuclei are absent from the decellularized samples, both in the lumen region and in the transverse region (Figure 2B,D). Masson’s trichrome staining results further show the absence of red/purple staining in DEM compared to that of native tissue (Figure 2E,F). This result implies that the cell nuclei was removed in DEM. At the same time, we observed that the Alcian blue staining remained in the DEM samples compared to native samples, which means that the connective matrix was maintained intact during the decellularization process (Figure 2G,H). Alcian blue staining further showed that the blue colors in the DEM and native samples did not have significant changes, which suggested that the carbohydrate-based glycosaminoglycan structures are preserved, and they appear to be intact in DEM when compared to the native tissue samples.

### 3.3. Immunohistochemistry Staining of Matrix

**Figure 3:**
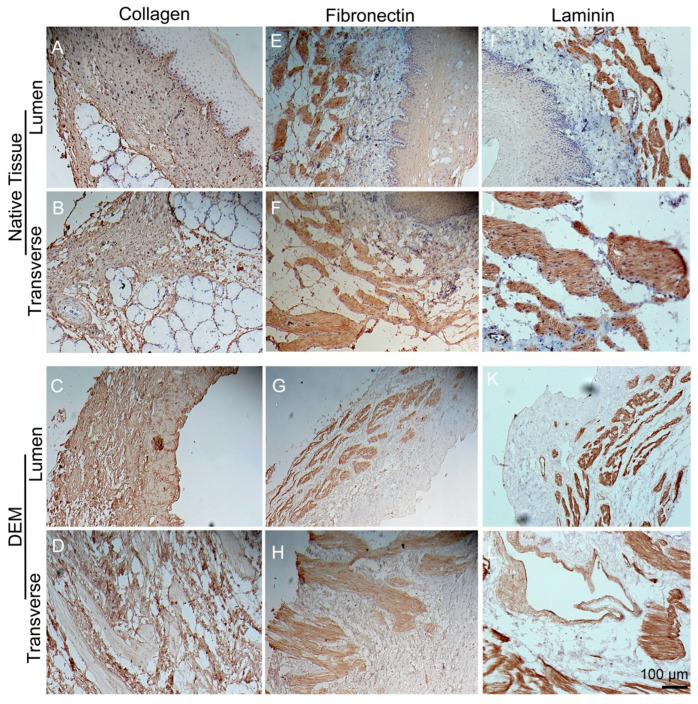
Immunohistochemical staining of matrix. IHC staining of collagen (**A**–**D**), fibronectin (**E**–**H**), and laminin (**I**–**L**) on the lumen side and transverse side of native porcine esophagus and DEM. Please see the detailed explanation for subfigures (**A**–**L**) in the above paragraph.

We performed immunohistochemical staining on the DEM and native tissue samples on lumen side and transverse side to qualitatively determine the intact appearance of the main matrix components: collagen, fibronectin, and laminin. From the staining results, collagen I/III in DEM (Figure 3C,D) shows similar structural patterns with a characteristic spindly form and preserved luminal indentations to the native samples (Figure 3A,B). Similarly, fibronectin stained in DEM (Figure 3G,H) also appeared to be intact and demonstrated quite a similarity to the native samples (Figure 3E,F) in terms of shape, texture, patterns, mosaic, even color and lightness. Finally, laminin in DEM (Figure 3K,L) has the location and appearance to match the native laminin tissue samples as the others (Figure 3I,J).

### 3.4. Compare Static Well Plate Culture to Dynamic Flow Culture Tissue Samples

**Figure 4:**
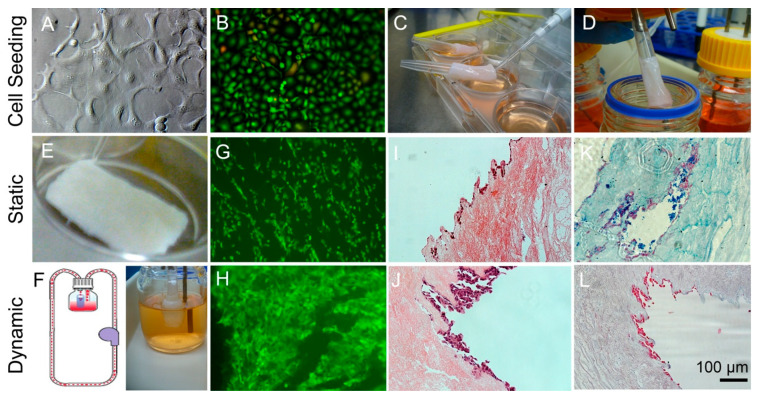
Cell viability in static and dynamic culture. (**A**) Light microscopy appearance of KYSE30 cell line. (**B**) Live/Dead of KYSE30 on hard plastic surface of well plate. (**C**) Cells seeding to lumen through perforated window in pipette tip. (**D**) Mounting esophagus to dynamic flow chamber. Comparison of static culture condition vs. dynamic condition in biocompatible scaffold: (**E**,**F**). Live/Dead staining on cells/matrix under static and dynamic culture (**G**,**H**). H&E staining (**I**,**J**). Alcian Blue staining on glycosaminoglycans (**K**,**L**).

To compare the cell morphologies on DEM under static well plate and dynamic culture systems, human ESCC cell line KYSE30 were seeded. The results show that KYSE30 seeded on the 2D well plate forms tight yet disorganized shapes as colonies of cells which may pile on each other after confluence and do not seem to slow cell growth rate even after reaching a confluence and myriad cell–cell contacts (Figure 4A). They attach well to stiff surfaces such as plastic well plates and rapidly expand cell numbers. On a well plate, they appear to spread out and confer quite a different morphology seemingly at random, disparate throughout the hard plastic surface (Figure 4B).

The cell viability staining result shows that KYSE30 cells seeded on DEM and placed in a well plate for 7 days (Figure 4E) qualitatively appear smaller and closer together (Figure 4G). There is no discernable ethidium homodimer staining, suggesting very few dead cells. H&E and Alcian Blue staining revealed cell positioning, morphological appearance, and matrix-related apparent interactions (Figure 4I,K). The cell seeding and appearance of continuous line along where the epithelium would be is thin in the static culture sample. There appears to be GAG residues in the static Alcian blue staining result (Figure 4K). The dynamic culture experiment was performed inside a 100 mL bioreactor perfused with culture media at 15 μL/min flow rate for 7 days. In order to seed KYSE30 cell line cancer cells successfully for the experiment in dynamic culture, it was found best to seed in a static condition for approximately 2 h first (Figure 4C); then, the cell-laden matrix was mounted onto the tip of a cut pipette (Figure 4D). The cells/matrix were cultured in a customized perfusion bioreactor (Figure 4F). KYSE30 cells seemed to proliferate and attach more closely in the dynamic culture experiment where they grew more rampant and seemed elongated compared with static culture (Figure 4H). The cells along the surface of the epithelium were frequently thicker in the dynamic culture (Figure 4J). The Alcian blue was free from GAG residue ‘debris’ (Figure 4L). There seemed to be less debris present, presumably due to the flow condition which cleared out the debris fragments and lingering particulates from the sample over time.

### 3.5. IHC Staining of Cancer Markers

**Figure 5:**
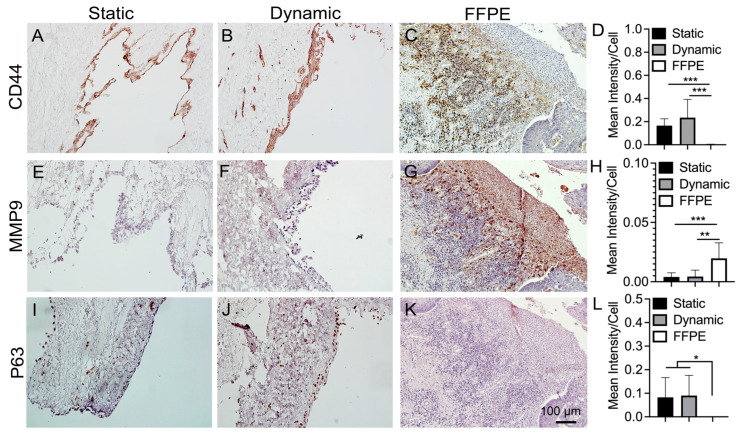
Immunohistochemical staining of CD44 (**A**–**C**), MMP9 (**E**–**G**), and P63 (**I**–**K**) on static, dynamic cultured samples and FFPE-ESCC samples. Please see the detailed explanation for subfigures (**A**–**C**,**E**–**G**,**I**–**K**) in the above paragraph. IHC staining on static vs. dynamic vs. clinical esophageal squamous cell carcinoma (ESCC). Semi-quantification of IHC staining per unit cell area for CD44 (**D**), MMP9 (**H**), and P63 (**L**) (n = 3). Asterisks show statistically significant difference between groups (*: *p* < 0.05, **: *p* < 0.01, and ***: *p* < 0.005).

To compare the expression level of cancer makers on KYSE30 cultured on the DEM under the two conditions, immunohistochemical staining was performed. At the same time, FFPE esophageal tumor biospecimens were stained to compare the similarity of cancer markers. IHC results show that KYSE30 cancer cells in both static and dynamic cultures express CD44 (Figure 5A,B), but FFPE-ESCC samples express CD44 on only a select cell population within the tissue biospecimen which weights against cell areas that do not express antibody markers in a semi-quantitative relationship (Figure 5C). The CD44 transmembrane receptor is multi-functional and varies based on exon splicing [37], widely associated with cell–cell interactions and migration with surface component cleavage in cancer migration [38,39]. The semi-quantitative result, which is quantified on a staining intensity/cell-area (the presence of cells in area which do not express any markers is weighted against cells in area which do express markers) basis, shows that KYSE30 expressed CD44 much more highly in the dynamic and static cultures, compared to FFPE-ESCC (Figure 5D), given that only some of the cells in the FFPE-ESCC samples expressed the CD44 marker, while all of the cells in the static and dynamic cultures expressed CD44. This result implied that the CD44 expressed on the FFPE-ESCC is heterogeneous. On the other hand, MMP9 expression only occurred in the FFPE-ESCC samples, which is much higher quantitatively (*p* < 0.05) compared to KYSE30 under static and dynamic cultures (Figure 5E–H). MMP9 is one of several tissue-remodeling enzymes capable of enzymatically breaking down ECM to facilitate cell migration, invasion, and wound healing, and is a surrogate marker for invasion of cancer cells [40]. There appears to be an interplay between CD44 and MMP9 expression (Figure 5C,G).

**Figure 6:**
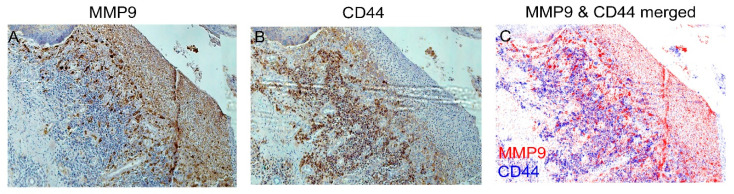
Qualitative interactions between CD44 and MMP9. CD44 expression (**A**) and MMP9 Expression (**B**) shown together using cover overlays on nearby tissue sections (**C**).

To further observe the distribution of staining of MMP9 and CD44 on FFPE-ESCC samples, two side-by-side comparisons taken from neighboring slide sections were stained using immunohistochemical staining, and the brown color was changed to either blue or red by adjusting hue to maintain contrast and brightness using Photoshop software, depending on where the section originated (blue for CD44 and red for MMP9). The slides were also rotated to an axis which gave the best comparison angle for viewing together. Two completely original slides stained separately were brought together as a single slide in the overlay from neighboring slides of the same tissue ESCC tumor biospecimen. They were overlaid together in the figure to show close interactions in the FFPE-ESCC tissue (Figure 6), which is not normally possible while using immunohistochemistry staining that only allows for one particular antibody to be displayed at a time. We employed this approach method as it was necessary to highlight the key apparent interactions within tissue biospecimen for easy viewing. We think CD44 interactions with MMP9 may be in part responsible for migration and remodeling through the tissue specimen. 

P63 as a transcription factor plays a role in development of squamous epithelium. P63 is localized to basement cells and may be overexpressed in skin-related cancer phenotypes [41]. Furthermore, P63 has implications in cell signaling, cell death, and cancer [42]. In both dynamic and static cultures, P63 was detected (Figure 5I,J). However, there seems to be a slightly stronger, albeit non-statistically significant, expression in the dynamic culture compared to static culture. Interestingly, none was detectable in the FFPE-ESCC samples (Figure 5L).

**Figure 7:**
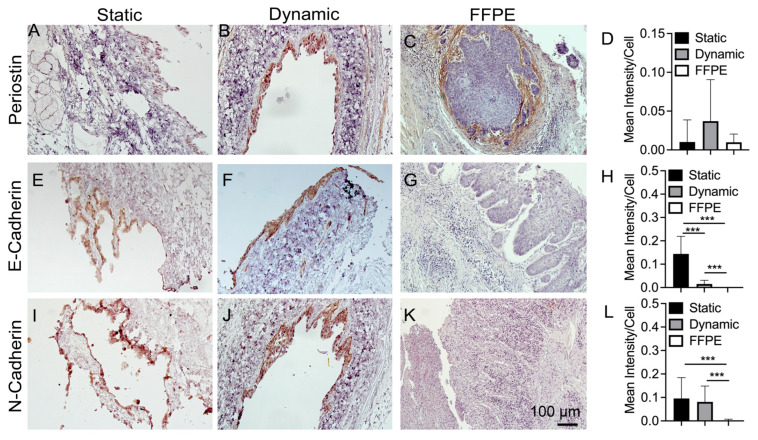
Immunohistochemical staining of periostin (**A**–**C**), E-cadherin (**E**–**G**), and N-cadherin (**I**–**K**) on static, dynamic cultured samples and FFPE-ESCC samples. Please see the detailed explanation for subfigures (**A**–**C**,**E**–**G**,**I**–**K**) in the above paragraph. IHC staining on static vs. dynamic vs. clinical esophageal squamous cell carcinoma (ESCC). Semi-quantification of IHC staining per unit cell area for periostin (**D**), E-cadherin (**H**), and N-cadherin (**L**) (n = 3). Asterisk “***” indicates statistically significant difference (*p* < 0.005).

Periostin is a matricellular protein belonging to a family of proteins which are shown to regulate key features of cancer behavior such as invasion, proliferation, remodeling, and dissemination [43]. Periostin as a cell-secreted protein may be upregulated for a wide range of cancers leading to cell survival, epithelial to mesenchymal transition (EMT), and angiogenesis [44], and may play a pivotal role in matrix cell signaling as a cytokine molecule related to EMT cues [45]. IHC results show that periostin appears to be expressed mainly in cells in the dynamic and static cultures (Figure 7A,B). Again, it seems like the dynamic culture expresses at moderately higher (0.04 staining intensity/cell-area vs. 0.01 intensity/area in static culture) levels (Figure 7D). In the FFPE-ESCC sample, the difference appears to be that periostin is mostly found in the matrix as opposed to being directly expressed by cells themselves (Figure 7C). KYSE30 cancer cells appear to express both E and N cadherins (Figure 7E,F,I,J). As with other static samples, the lack of flow rate as in dynamic culture seems to lead to excess appearance of debris/particulates around the sample (Figure 7I). It was interesting to note that the FFPE-ESCC does not appear to express either N or E cadherins (Figure 7G,K). The E-cadherin exhibits higher expression in static culture than dynamic condition (*p* < 0.005) (Figure 7H). However, N-cadherin semi-quantitative expression was similar for static and dynamic cultures (*p* > 0.05) (Figure 7L). E and N cadherin expression is considered one of the hallmarks for an EMT, as E-cadherin is lost while N-cadherin is upregulated [46]; thus, it is a natural target for investigation when considering cancer cell migration and behavior via antibody interactions. It is implied from this result that the dynamic culture may facilitate the loss of E-cadherins and upregulate the mesenchymal transition for metastasizing cell behavior.

**Figure 8:**
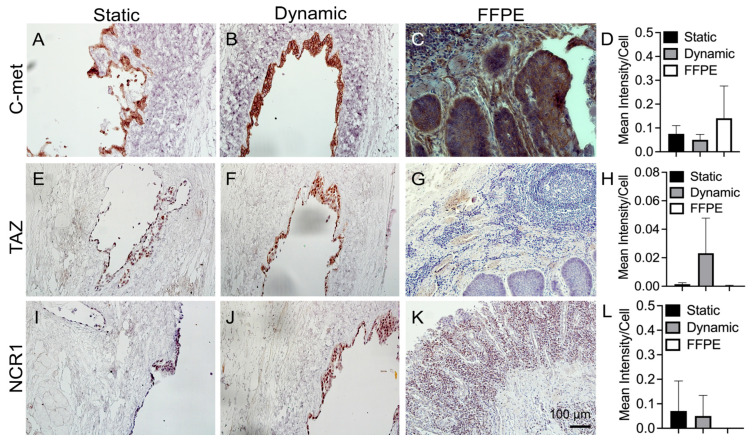
Immunohistochemical staining of C-met (**A**–**C**), TAZ (**E**–**G**), and NCR1 (**I**–**K**) on static, dynamic cultured samples and FFPE-ESCC samples. Please see the detailed explanation for subfigures (**A**–**C**,**E**–**G**,**I**–**K**) in the above paragraph. IHC staining on static vs. dynamic vs. clinical esophageal squamous cell carcinoma (ESCC). Semi-quantification of IHC staining per unit cell area for C-met (**D**), TAZ (**H**), and NCR1 (**L**) (n = 3).

C-met expressed in all samples (Figure 8A–C) and their relative or semi-quantitative results show there is no significant difference (*p* > 0.05) (Figure 8D). C-met is associated with cell scattering, survival, and proliferation, and is related to EMT as it may be genetically altered in association with cancer progression and metastasis [47]. C-met overexpression was a predictor of poor prognosis in certain types of cancer [48]. TAZ, like our other IHC targets, is implicated in differentiation, apoptosis, invasion, migration, EMT, and ECM stiffness [49]. TAZ expression gives information pertaining to the ECM, implicated in directing fibrosis, fibroblast activation, and production of ECM proteins, and because of its role in EMT contributing to cancer stemness [50]. Results show that TAZ was not expressed in the FFPE-ESCC samples (Figure 8G), but strongly expressed in the dynamic culture (Figure 8F) and lightly in the static culture (Figure 8E). Semi-quantitative positive density per unit cells given by Figure 8H indicates that the KYSE30 cultured in the dynamic condition expressed higher TAZ compared to static culture and FFPE-ESCC samples, although there is no statistical difference between them. It is reasonable to suspect that the higher TAZ expression in dynamic cultures may result from the fluid flow condition. 

The natural cytotoxicity receptor (NCR1) lineage is involved with activating NK cell cytotoxicity in response to tumor and viral ligand expression [51]. We chose NCR1 expression to evaluate whether cancer cells are expressing for possible NK-mediated cytotoxicity or whether it may be suppressed. NCR1 does not appear in the FFPE-ESCC samples (Figure 8K). It does however make an appearance in the static and dynamic culture samples of KYSE30 cell line (Figure 8I,J). Semi-quantitative result shows that there is no significant difference in the three groups (*p* > 0.05) (Figure 8L).

## 4. Discussion

In this study, we developed a new decellularization method to decellularize porcine esophagus rapidly for use in the development of an esophageal cancer model. Porcine esophagus is similar to human esophagus, and cancer cell behavior as it interacts with originally living esophageal matrix may recognize, attach, modify, and experience a familiar environment, which may lead to observing phenomena that may not be found in other synthetic or altered substrate-based esophageal cancer models. The decellularization method was improved, as it brought the ease with which the epithelial layer was detached from the submucosa layer, and allowed complete cell removal without appearing to damage the matrix. This is a significant improvement over the conventional decellularization of such tissues because it removes the majority of epithelial cells in a single step without using any detergents. Most conventional decellularization methods could potentially denature resulting matrix properties [52,53], which may lead to results less likely to reflect in vivo conditions. To our knowledge, this is the first report of a rapid, detergent-free approach for tissue decellularization which can remove nearly all cell nuclei [54]. 

Cell studies show that our DEM is biocompatible, on which cancer cells proliferated well. The KYSE30 cancer cells seeded on the decellularized matrix were able to rapidly divide, occasionally disseminated throughout the matrix as they are apt to colonize within the matrix. In this study, we also successfully developed a dynamic culture system using a customized bioreactor. This system was easy to set up to perfuse culture media for cells, which provides a dynamic supply of nutrition to cells for proliferation and waste removal. The constant flow of nutritional supply and waste clearance maintained the cell growth expansion rate. This dynamic flow culture mode allowed cells to grow in higher density more efficiently. Dynamic culture also provides a large surface area for extended growth of cell populations more than a typical static culture well plate experiment. Additionally, the benefit of this dynamic culture system is that it can provide longer culture durations without maintenance intervention, since cell culture media is perfused continuously via a predetermined container volume and may be set up in advance for extended experiment conditions in order to gradually grow a larger tumor tissue than would otherwise be possible. Thus, this dynamic culture system brings promising potential to develop a biomimetic esophageal cancer model, mimicking an in vivo esophageal cancer tissue-growing microenvironment in the future. 

The FFPE-ESCC samples revealed cell phenotypical expression patterns not always seen in the static or dynamic DEM cultures (Figure 5, Figure 6, Figure 7 and Figure 8). CD44 is known to play a role in cell invasiveness due to cleavage by metalloprotease enzyme during migration and is considered as a possibility for targeted therapy [55]. In FFPE-ESCC tumor samples, wherever there is a strong appearance of MMP9, the CD44 is diminished greatly or nonexistent while appearing highly expressed just nearby (Figure 6C). It may be that dense CD44 expression precludes metastasis or tissue remodeling via sequestration of MMP9 protease prior to activation, possibly directing cell specific signals towards a specific site of the ECM for invasion. This immunohistochemical appearance of reciprocation (Figure 6A–C) between CD44 and MMP9 [39,56,57,58,59,60] was not expected to exhibit in the FFPE-ESCC sample and its significance remains to be clarified in future experiments where we can investigate an underlying mechanism for invasion and matrix remodeling expression interplay in a DEM sample to simulate the clinical tissue. Although the KYSE30 in DEM did not express MMP9, there was a significant difference in the expression of CD44 between FFPE-ESCC and KYSE30/DEM, because all cells in the DEM samples expressed CD44, whereas only part of what are presumed to be disseminating cells involved with MMP9 signaling expressed CD44 in the FFPE-ESCC tumor tissue samples. 

For the matrix-relevant protein periostin, both FFPE-ESCC biospecimens and DEM samples exhibited periostin expression. This result implies that periostin, as an extracellular secretion protein [61], may be related to ESCC cell invasion [62]. For another protein receptor tyrosine kinase, c-met, which is expressed on the surface of many cells [63] and overexpressed in esophageal squamous cell carcinoma [64], both FFPE-ESCC tumor and DEM samples expressed this protein at similar levels. 

For the semi-quantitative data, it is noted that at least eight immunohistochemistry pictures taken of multiple ROI were evaluated for each group and their staining intensity/cell-area was averaged together for each quantitative relationship graph; thus, the representative images in Figure 5, Figure 6, Figure 7 and Figure 8 may not fully adhere to the graphed results as they are merely some of many pictures used to generate the statistical calculation.

Using a cell line such as KYSE30 to perform single cell culture experiments in a decellularized esophageal matrix has some potential drawbacks [65]. Cells often depend on cell–cell interactions [66,67,68,69] to enable features such as expression of matrix metalloproteins [70] and to signal for excretion of substrates and ligands or for splicing exosomes to produce protein modifications [39,55]. Furthermore, KYSE30 is rendered immortal [71,72,73] via transfection pathway unrelated to its original clinically definable features from an originating cancer and has had a chance to evolve [74] over time as a cell line with unstable, ablated chromosomal features [75], all of which impact behavior regardless onto which ECM or matrix it is placed. In future experiments, it will be desirable to culture patient-derived tumor cells in the presence of additional accessory cell counterparts such as mesenchymal cells [76,77] for a better cancer model. Although we did not observe significant cell migration into the DEM possibly due to density or stiffness as is [78], the porcine esophagus-derived matrix demonstrated an excellent property to support cancer cell growth, possible matrix remodeling, and cell invasion. Another limitation of this study is that the culture period was too short to allow for any extended viewing of long-term behaviors in the perfusion bioreactor, as the long duration potentially increased the risk of contamination. For longer culture, an easier culture medium change protocol may need to be creatively developed.

Thus, this is the first step for our long-term goal of building a dynamic culture system and decellularization method for esophageal cancer model in vitro. In the future, we may co-culture with multiple cell compartments. It would be valuable to develop an advanced biomimetic esophageal tumor model using the DEM and dynamic culture system developed in this study with other cells for cancer cell progression, invasion, and metastasis mechanistic studies.

## 5. Conclusions

In this study, we developed a new in vitro model of esophageal cancer using a detergent-free decellularized porcine esophageal matrix in a customized bioreactor. The new decellularization method can effectively remove cells and qualitatively maintain matrix components. The quantitative DNA experiment indicated successful decellularization and DNA removal. The decellularized matrix supported KYSE30 cancer cells to proliferate well in the static well plate and dynamic bioreactor. The esophageal cancer KYSE30 cells cultured on the DEM in the dynamic condition expressed different levels of cancer marker expression, compared to those in the static culture condition, but some similarities appeared compared to the FFPE-ESCC biospecimens. These findings bring new potential to using the decellularized matrix for future experiments in studying cancer behavior and practical applications for esophageal cancer models, as well as non-cancer specific tissue repair.

## Data Availability

Available upon request.

## Supplementary Materials

The following supporting information can be downloaded at: https://www.mdpi.com/article/10.3390/bioengineering10010096/s1. The original code for generating semi-quantitative data for slides taken of ROI data [79].
