# Supplementary material for: A New Model of Esophageal Cancers by Using a Detergent-Free Decellularized Matrix in a Perfusion Bioreactor
Source: Bioengineering (Basel). 2023 Jan 11;10(1):96. doi: 10.3390/bioengineering10010096 (PMC9854977; doi:10.3390/bioengineering10010096)
Supplement: Supplementary file 1 [file bioengineering-10-00096-s001.zip › bioengineering-2135179-supplementary.pdf]

A new model of esophageal cancers by using a detergent-free decellularized matrix in a perfusion bioreactor

Jordan Brennan, Michael L. Lu and Yunqing Kang

Supplementary material:

Code for semi-quantitative image processing.

Based on the reference [1] and with NIH ImageJ Software, a customized code was written to generate semi-quantitative Data for slides taken of ROI data:

```
extension = ".jpg";
dir1 = getDirectory("Choose Source Directory ");
//dir2 = getDirectory("Choose Destination Directory ");
setBatchMode(true);
n = 0;
processFolder(dir1);

function processFolder(dir1) {
  list = getFileList(dir1);
  for (i=0; i<list.length; i++) {
      if (endsWith(list[i], "/"))
                processFolder(dir1+list[i]);
            else if (endsWith(list[i], extension))
    processImage(dir1, list[i]);
  }
}
function processImage(dir1, name) {
   open(dir1+name);
   print(n++, name);
   // below is code to analyze and process the image
 open(dir1+name);
 run("Colour Deconvolution2", "vectors=[H DAB] output=8bit_Transmittance simulated cross hide");
 selectWindow(name+"-(Colour_3)");
 selectWindow(name+"-(Colour_2)");
 setAutoThreshold("Default no-reset");
 setThreshold(0, 117);
 setOption("BlackBackground", false);
 run("Convert to Mask");
 run("Measure");
 selectWindow(name+"-(Colour_1)");
 setAutoThreshold("Default no-reset");
 setThreshold(0, 114);
 run("Convert to Mask");
 run("Watershed");
```

```
run("Analyze Particles...", "size=19.50-26.00 exclude summarize");
 //saveAs(extension, dir2+name);
  }
```
